# Genome-wide survey and identification of AP2/ERF genes involved in shoot and leaf development in *Liriodendron chinense*

**DOI:** 10.1186/s12864-021-08119-7

**Published:** 2021-11-08

**Authors:** Yaxian Zong, Ziyuan Hao, Zhonghua Tu, Yufang Shen, Chengge Zhang, Shaoying Wen, Lichun Yang, Jikai Ma, Huogen Li

**Affiliations:** Co-Innovation Center for Sustainable Forestry in Southern China, Key Laboratory of Forest Genetics and Biotechnology of Ministry of Education, Nanjing Forestry University, Nanjing, 210037 China

**Keywords:** *Liriodendron chinense*, AP2/ERF family, Transcription factors, Shoot-specific, Leaf development

## Abstract

**Background:**

*Liriodendron chinense* is a distinctive ornamental tree species due to its unique leaves and tulip-like flowers. The discovery of genes involved in leaf development and morphogenesis is critical for uncovering the underlying genetic basis of these traits. Genes in the AP2/ERF family are recognized as plant-specific transcription factors that contribute to plant growth, hormone-induced development, ethylene response factors, and stress responses.

**Results:**

In this study, we identified 104 putative AP2/ERF genes in the recently released *L. chinense* genome and transcriptome database. In addition, all 104 genes were grouped into four subfamilies, the AP2, ERF, RAV, and Soloist subfamilies. This classification was further supported by the results of gene structure and conserved motif analyses. Intriguingly, after application of a series test of cluster analysis, three AP2 genes, *LcERF 94*, *LcERF 96,* and *LcERF 98*, were identified as tissue-specific in buds based on the expression profiles of various tissues. These results were further validated via RT-qPCR assays and were highly consistent with the STC analysis. We further investigated the dynamic changes of immature leaves by dissecting fresh shoots into seven discontinuous periods, which were empirically identified as shoot apical meristem (SAM), leaf primordia and tender leaf developmental stages according to the anatomic structure. Subsequently, these three candidates were highly expressed in SAM and leaf primordia but rarely in tender leaves, indicating that they were mainly involved in early leaf development and morphogenesis. Moreover, these three genes displayed nuclear subcellular localizations through the transient transformation of tobacco epidermal cells.

**Conclusions:**

Overall, we identified 104 AP2/ERF family members at the genome-wide level and discerned three candidate genes that might participate in the development and morphogenesis of the leaf primordium in *L. chinense*.

**Supplementary Information:**

The online version contains supplementary material available at 10.1186/s12864-021-08119-7.

## Background

Plant morphogenesis is mainly related to the shoot as well as the activity of the shoot apical meristem (SAM) [1, 2], which further gives rise to stems, tender leaves, or other tissues and organs. Plant endogenous hormones are recognized to play a crucial role in regulating leaf development and morphogenesis [3, 4]. Moreover, functional dissections of plant transcription factors (TFs), such as NAC [5], KNOX [6], and AP2/ERF [7, 8], which have been largely described as being involved in shoot and leaf development, have been revealed from studies on *Arabidopsis*.

APETALA2/ethylene responsive element-binding proteins (AP2/EREBPs) are a well-known transcription family that has been reported to be involved in ethylene response, biotic or abiotic stress resistance, cell differentiation, cell expansion, and stress signaling pathways in plants [9–11]. As one of the largest TF families of plants, the AP2/ERF family is composed of four subfamilies with over 100 members in various taxa [12–14]. A feature of the AP2/ERF-type DNA binding domain, which consists of 60 ~ 70 residues, is universally contained in the proteins of this family [15]. Moreover, on the basis of the types and quantities of conserved domains, the AP2/ERF family can be divided into the AP2, RAV, ERF, and Soloist subfamilies. Proteins of the AP2 subfamily contain two repetitive AP2 domains, while the ERF subfamily proteins have only a single AP2 domain. In the RAV subfamily, tandem AP2 and B3 domains were found in the primary protein sequences [16–18]. Finally, the Soloist subfamily has been historically regarded as an ERF member and is currently regrouped into a novel subfamily due to its single AP2 domain and strong sequence divergence [16, 19]. In addition, based on the sequence similarity of the AP2/ERF motifs, the ERF family is further divided into the ERF and DREB subfamilies [18, 20].

Although four clades have been maintained in the AP2/ERF family, their functions largely depend on the interaction of their motifs with specific regulatory elements [16]. In general, the ERF and DREB subfamilies primarily function in resistance to biotic or abiotic stresses [21], and the “WLG” motif is considered a typical feature of these subfamilies [18]. Additionally, the DREB subfamily is mainly involved in improving abiotic stress tolerance in plants, including salinity stress, drought stress, water deficit, low temperature, and other abiotic stresses, via the interaction of a core motif of A/GCCGAC with downstream dehydration responsive elements (DREs) [22]. The ERF subfamily participates in defense by integrating the cis-acting element AGCCGCC with its GCC box [23, 24]. However, this is not always the case, and this resistance can be interrupted by the VIII groups of these families [25]. *DRN* and *DRNL* genes in Arabidopsis hierarchically interact in auxin signaling and patterning of the apical embryo. In addition, *LEAFY PETIOLE (LEP)* acts as a positive regulator of gibberellic acid-induced germination and is involved in the formation of petioles [26]. The AP2 subfamily usually regulates the development of shoots as well as the stem cell niche during embryonic pattern formation [27, 28]. In summary, these inferences from previous studies provide direction for comprehensively understanding AP2/ERFs and the discovery of novel genes involved in leaf and shoot development.

*Liriodendron chinense* (Hemsl.) Sarg. is a relict tree species that is native to southern China. It is famous for its odd leaf shape, which looks like a “riding jacket”, and is widely used as an ornamental in landscapes and gardens. However, the underlying genetic mechanisms of leaf development and morphogenesis remain poorly understood. As a valuable ornamental plant, it is meaningful to understand the development and morphogenesis of *L. chinense* leaves. Along with the recent release of the *L. chinense* genome and transcriptome information [29–31], we have new insights for investigating gene families and exploring candidate genes involved in SAM and leaf development.

In this study, we identified 104 LcAP2/ERF genes by combining a genome-wide survey and transcription data of various tissues. Moreover, we described the conserved motif, gene structure, and phylogenetic analyses and divided the LcAP2/ERF genes into four subfamilies of approximately 14 groups. Through expression profile analysis and series test of cluster (STC) analysis, we discovered the genes expressed specifically in shoots and further examined the expression patterns of these candidates dynamically in the different developmental stages. This work will lay a foundation for the comprehensive understanding of the LcAP2/ERF family and will also be helpful for determining candidate genes involved in leaf development in *L. chinense*.

## Results

### Identification of AP2/ERF TFs in *L. chinense*

Based on the HMM profiles (PF00847) and homology searches, a total of 104 putative AP2/ERF genes designated *LcERF1* to *LcERF104* were identified in *L. chinense*. All these candidates contained one or more AP2/ERF domains according to conservative domain analysis. Then, we described the characteristics of their proteins, including the coding sequence (CDS) length, protein length, molecular weight (MW), isoelectric point (PI), and predicted subcellular localization (see Additional file 1: Table S1). Accordingly, the protein lengths of these 104 AP2/ERFs ranged from 100 aa (*LcERF29*) to 758 aa (*LcERF42*), with an average of approximately 317 aa (Table 1). Moreover, the molecular weights of the proteins varied from 11.48 kDa (*LcERF29*) to 84.42 KDa (*LcERF42*). In addition, the isoelectric point values of these proteins ranged from 4.72 (*LcERF27*) to 10.22 (*LcERF67*). The subcellular localization predicted that 83 LcERF proteins were located in the nuclear region, 13 LcERF proteins were located in the chloroplast region, and the remaining genes were distributed in the cytoplasm, mitochondria, plasma membrane, and other areas (see Additional file 1: Table S1).

**Table 1:** List of the 104 *AP2/ERF* genes identified in *Liriodendron Chinense*

Gene name	Gene ID	Location	Protein length (aa)	Introns	Family group
LcERF1	Unigene40981_All	Scaffold211	261	0	I
LcERF2	Lchi03057	Scaffold506	328	1	I
LcERF3	Lchi07965	Scaffold708	325	1	I
LcERF4	Lchi07966	Scaffold708	561	2	I
LcERF5	Lchi22931	Scaffold1519	432	1	I
LcERF6	Lchi09796	Scaffold2048	316	1	I
LcERF7	Lchi16995	Scaffold3097	316	0	I
LcERF8	Lchi23250	Scaffold142	152	0	II
LcERF9	Lchi16170	Scaffold408	193	1	II
LcERF10	Unigene12650_All	Scaffold416	188	0	II
LcERF11	Lchi16911	Scaffold480	199	1	II
LcERF12	Unigene40401_All	Scaffold525	185	0	II
LcERF13	Unigene20830_All	Scaffold836	186	0	II
LcERF14	Lchi11957	Scaffold345	224	1	III
LcERF15	Lchi04946	Scaffold530	235	1	III
LcERF16	Lchi04947	Scaffold530	295	1	III
LcERF17	CL2522.Contig2_All	Scaffold530	223	0	III
LcERF18	CL10877.Contig3_All	Scaffold530	223	0	III
LcERF19	Unigene6126_All	Scaffold530	211	0	III
LcERF20	Lchi33109	Scaffold1203	229	1	III
LcERF21	Lchi33111	Scaffold1203	227	1	III
LcERF22	Lchi34895	Scaffold1374	513	4	III
LcERF23	Lchi29925	Scaffold1675	425	3	III
LcERF24	Lchi08587	Scaffold39	420	1	III
LcERF25	CL5589.Contig2_All	Scaffold345	203	0	III
LcERF26	Unigene11386_All	Scaffold432	211	0	III
LcERF27	CL5589.Contig1_All	Scaffold530	246	0	III
LcERF28	Lchi00950	Scaffold723	217	0	III
LcERF29	Lchi01616	Scaffold1191	100	1	III
LcERF30	Unigene5530_All	Scaffold1191	252	0	III
LcERF31	Lchi32377	Scaffold1289	210	0	III
LcERF32	Lchi26370	Scaffold1364	193	1	III
LcERF33	Lchi08922	Scaffold3419	244	0	III
LcERF34	Lchi28169	Scaffold654	418	1	IV
LcERF35	Lchi23878	Scaffold1043	141	0	IV
LcERF36	Lchi22387	Scaffold1263	229	1	IV
LcERF37	Lchi13652	Scaffold1315	429	1	IV
LcERF38	Lchi30363	Scaffold2365	475	3	IV
LcERF39	Lchi30365	Scaffold2365	354	1	IV
LcERF40	Lchi31374	Scaffold3032	404	1	IV
LcERF41	Lchi34724	Scaffold3708	355	1	IV
LcERF42	Lchi10868	Scaffold159	758	2	V
LcERF43	Lchi11945	Scaffold345	421	7	V
LcERF44	Lchi25937	Scaffold1371	183	1	V
LcERF45	Lchi16637	Scaffold2432	226	1	V
LcERF46	Lchi34468	Scaffold2926	206	1	V
LcERF47	Lchi05084	Scaffold3476	136	1	V
LcERF48	Lchi07311	Scaffold172	268	0	VI
LcERF49	Lchi22103	Scaffold920	369	2	VI
LcERF50	Lchi17039	Scaffold3097	359	1	VI
LcERF51	Lchi02638	Scaffold416	310	1	VII
LcERF52	Lchi02639	Scaffold416	362	1	VII
LcERF53	Lchi11452	Scaffold525	383	1	VII
LcERF54	Lchi04620	Scaffold775	290	1	VII
LcERF55	Lchi04621	Scaffold775	289	1	VII
LcERF56	Lchi04623	Scaffold775	236	1	VII
LcERF57	Lchi07083	Scaffold135	273	1	VIII
LcERF58	Lchi07084	Scaffold135	203	0	VIII
LcERF59	Lchi13371	Scaffold1075	207	1	VIII
LcERF60	Lchi11824	Scaffold1130	375	3	VIII
LcERF61	Lchi13392	Scaffold1763	207	1	VIII
LcERF62	Unigene7795_All	Scaffold1763	205	0	VIII
LcERF63	Lchi31572	Scaffold1784	180	1	VIII
LcERF64	Lchi08484	Scaffold39	204	1	IX
LcERF65	Lchi09908	Scaffold79	316	1	IX
LcERF66	Unigene24905_All	Scaffold79	170	0	IX
LcERF67	Lchi01406	Scaffold432	211	1	IX
LcERF68	Unigene35921_All	Scaffold432	301	0	IX
LcERF69	Lchi08172	Scaffold580	324	0	IX
LcERF70	Lchi07909	Scaffold708	174	1	IX
LcERF71	Lchi31530	Scaffold803	105	2	IX
LcERF72	CL9762.Contig1_All	Scaffold1024	307	0	IX
LcERF73	Lchi05992	Scaffold1024	250	1	IX
LcERF74	Lchi05993	Scaffold1024	350	2	IX
LcERF75	Lchi26525	Scaffold1934	272	1	IX
LcERF76	Lchi26532	Scaffold1934	361	3	IX
LcERF77	Lchi28702	Scaffold54	309	2	X
LcERF78	Unigene10666_All	Scaffold100	235	1	X
LcERF79	Lchi02215	Scaffold682	229	1	X
LcERF80	Lchi02216	Scaffold682	196	1	X
LcERF81	Lchi18461	Scaffold943	403	1	X
LcERF82	Lchi11856	Scaffold1130	128	1	X
LcERF83	Lchi01932	Scaffold1191	292	1	X
LcERF84	Lchi20453	Scaffold1167	328	2	VI-L
LcERF85	Lchi14855	Scaffold41	394	6	AP2
LcERF86	Lchi23120	Scaffold192	405	7	AP2
LcERF87	Lchi16948	Scaffold480	550	9	AP2
LcERF88	Lchi08043	Scaffold502	680	6	AP2
LcERF89	Lchi11241	Scaffold503	375	8	AP2
LcERF90	Lchi28881	Scaffold509	474	7	AP2
LcERF91	Lchi06162	Scaffold527	524	7	AP2
LcERF92	Lchi03252	Scaffold764	535	6	AP2
LcERF93	CL7987.Contig2_All	Scaffold805	572	12	AP2
LcERF94	Unigene5404_All	Scaffold2118	490	6	AP2
LcERF95	Lchi33401	Scaffold2225	563	6	AP2
LcERF96	Lchi13837	Scaffold2467	662	7	AP2
LcERF97	CL6967.Contig2_All	Scaffold2956	327	6	AP2
LcERF98	Unigene39546_All	Scaffold3476	468	7	AP2
LcERF99	Lchi08779	Scaffold67	376	1	RAV
LcERF100	Lchi02516	Scaffold100	607	4	RAV
LcERF101	Lchi02519	Scaffold100	428	2	RAV
LcERF102	Lchi15640	Scaffold1242	354	2	RAV
LcERF103	Lchi23744	Scaffold1330	361	1	RAV
LcERF104	Lchi32356	Scaffold3563	235	5	Soloist

### Phylogenetic analysis and classification of LcERF genes

**Fig. 1:**
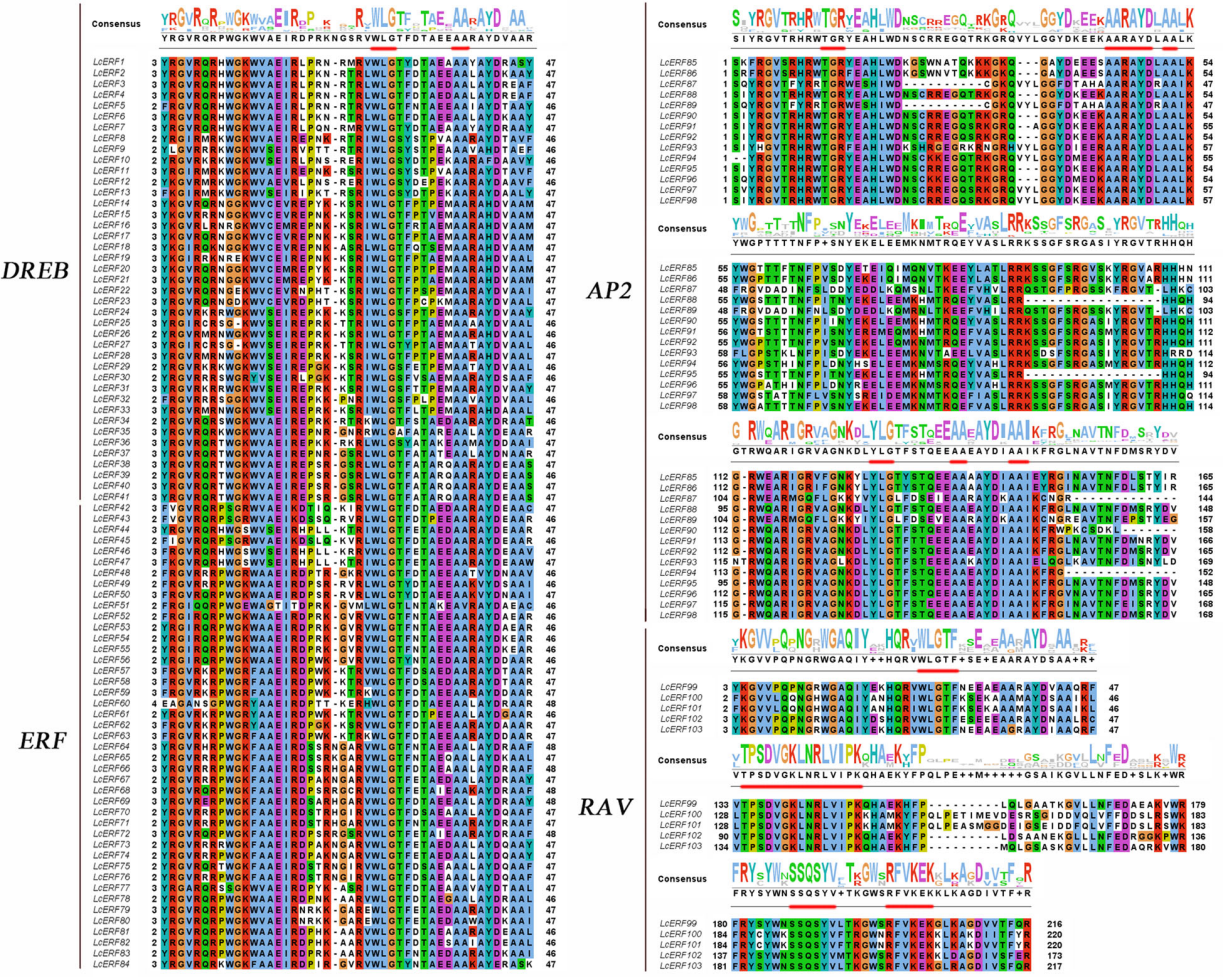
Visualization of LcAP2/ERF conserved domain. The amino acid sequences of AP2 domains from 104 AP2/ERF proteins were aligned by ClustalW. Among them, the DREB and ERF subfamilies all retain one AP2 domain that contains WLG, AA and other motifs. The AP2 subfamily retains two AP2 domains that contain TGR, RAYD, YLG and other motifs. In addition, the RAV subfamily retains an AP2 domain and a B3 domain. The bold-red lines display the unique motifs of the AP2/ERF family

Based on conservative domain analysis and multiple alignments of LcERF protein sequences, the 104 LcERF proteins were categorized into four subfamilies, including the ERF, AP2, RAV, and Soloist subfamilies. All 84 ERF proteins contained a single AP2 domain, and based on the characteristics of the amino acid sequences and domains that they encode, these genes were further divided into two subfamilies, which were named the DREB and ERF subfamilies and covered 41 and 43 members, respectively. However, among the remaining genes, 14 genes were identified as members of the AP2 family owing to their tandemly repeated double AP2/ERF domain. In addition, 5 genes that not only possessed a single AP2/ERF domain but also displayed a B3 domain were classified in the RAV subfamily. The last one, *LcERF104*, is homologous to the Arabidopsis Soloist gene (*At4g13040*) and was classified in the Soloist subfamily. According to the description in Nakano’s study [19], the DREB subfamily comprises four parts, named I, II, III, and IV, which contain 7, 6, 20, and 8 members, respectively. The ERF subfamily genes can be divided into seven groups based on phylogenetic analysis and belong to the V, VI, VII, VIII, IX, X, and VI-L parts with 6, 3, 6, 7, 13, 7, and 1 members, respectively. The sequence alignment of LcERF proteins showed that the WLG element was highly conserved in the ERF, DREB and RAV subfamilies but less conserved in the AP2 subfamily. However, RAYD, AA, and other elements were conserved in the AP2 subfamily (Fig. 1).

**Fig. 2:**
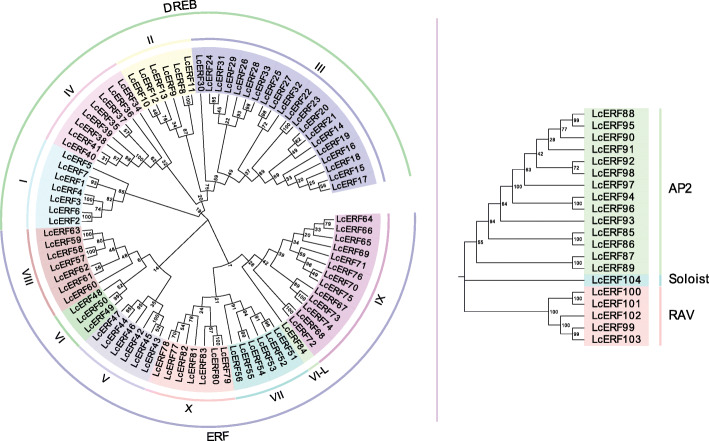
Phylogenetic tree using the NJ method displaying the relationships among the AP2/ERF family of *L. chinense*. The phylogenetic tree was constructed using AP2/ERF domain sequences by the NJ method with 1000 bootstrap replicates in MEGA 7.0. The evolutionary distances were computed using the Poisson model. All 104 LcERF proteins were categorized into four subfamilies, including the ERF, AP2, RAV, and Soloist subfamilies. The ERF subfamily members were further divided into DREB subfamily (Groups I, II, III, IV) and ERF subfamily (Groups V, VI, VII, VIII, IX, X, VI-L)

The evolutionary relationships of all candidate genes were further illustrated by phylogenetic analysis. According to the unrooted tree profile, AP2, RAV, and Soloist were clustered in a separate branch within the subfamily. However, ERF subfamily were divided into 2 large branches, the ERF branch and the DREB branch, and the ERF and DREB branches were divided into 7 and 4 groups, respectively (Fig. 2). Moreover, these findings coincided with the grouping of the ERF subfamily described above based on the conserved motifs (Fig. 1). In addition, this result showed the same clustering pattern as that obtained by the classification method based on alignment with Arabidopsis (Table 1). As a result, we propose that these 104 putative genes are indeed AP2/EFR family genes in *L. chinense*.

### Gene structure and conserved motif analysis

**Fig. 3:**
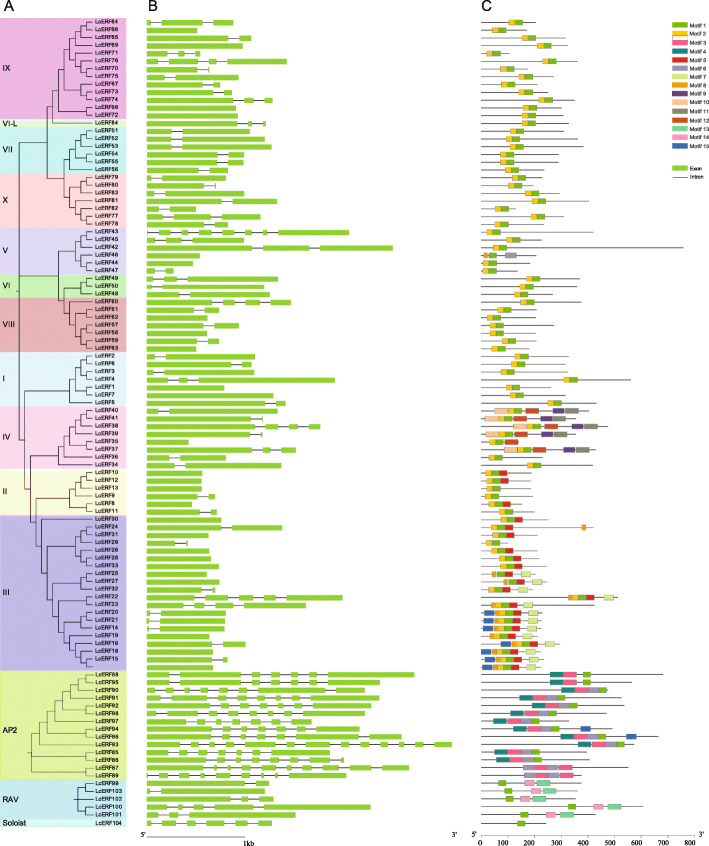
Gene structure and conserved motif analysis of 104 LcERF genes. (A) Phylogenetic tree of 104 LcERF proteins. (B) Exon-intron structures of LcERF genes. Green boxes represent exons, and black lines represent introns. (C) Conserved motifs of *LcERF* proteins. The motifs, numbers 1–15, are displayed in different colored boxes

To further understand the structural composition of LcERF genes, we analyzed the genomic DNA sequence using the online Gene Structure Display Server, with the locations of exons and introns provided by the *L. chinense* genomic resource. According to the structural characteristics of LcERF genes, the number of introns varied among the distinct subfamilies (Fig. 3A). Except for a few members carrying more than one intron, most of the DREB and ERF subfamily genes had only one intron or even no introns in their genomic DNA. In the AP2 subfamily, all the genes possessed numerous introns, with intron numbers ranging from 6 to 12. Furthermore, *LcERF93* was considered to have the most introns with 12, even though most *AP2* genes contained 6 or 7 introns. Moreover, four of the five RAV members possessed one or two introns, and the single Soloist member contained five introns (Fig. 3B). In addition, the position of introns also presented interesting differences among different subfamilies. Concerning the sequences with an intron, the position of their intron was mostly near the N-terminus or C-terminus and rarely in the middle of the sequence because these sequences usually consisted of a long and a very short exon. In general, the members with close evolutionary relationships and from the same subfamily had similar exon and intron structures in terms of intron number and position and exon length.

Conserved motifs of 104 LcERF genes were identified using the MEME (Multiple Em for Motif Elicitation) tool. A total of 15 conserved motifs were displayed in the 104 LcERF proteins (Fig. 3C). The amino acid length of the 15 motifs ranged from 15 to 50. Even though all of them had the AP2 domain, the protein sequences of the domain differed between the ERF and AP2 subfamilies. As AP2 DNA-binding motifs, motif 1 and motif 2 joined together and appeared in both the DREB and ERF subfamilies, except for special cases of motif 2, which also existed independently in the RAV and Soloist subfamilies. In the AP2 subfamily, the AP2 domain consisted of motif 3 and motif 6, and some of them also contained motif 4 and motif 12. In addition, although most of the ERF subfamily members shared the two conserved motifs of motif 1 and motif 2, the other motifs varied in the different proteins. In the DREB subfamily, the proteins contained relatively more conserved motifs than in the ERF subfamily, especially in group III and group IV. Motifs 5, 7, 8, and 15 were detected in some group III proteins, and motifs 9, 10, 11, and 12 were found in most group IV members. Moreover, motifs 13 and 14 only existed in the B3 domain and were also considered specific to the RAV subfamily.

### Expression profiles of LcERF genes in different tissues

**Fig. 4:**
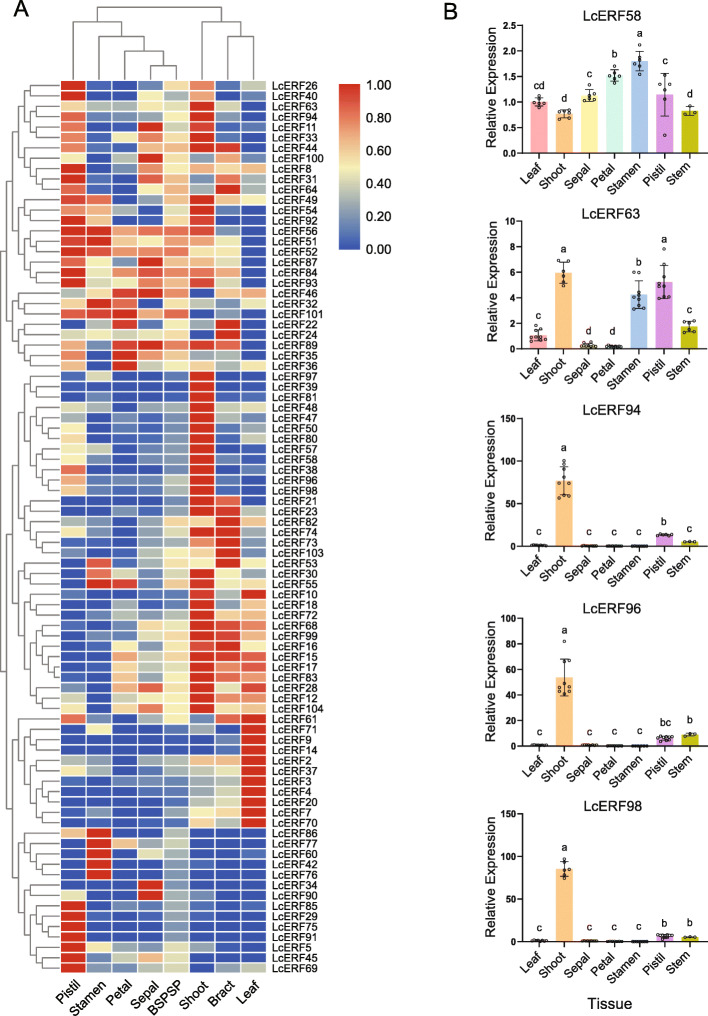
Expression patterns of LcERFs in different tissues. (**A**) Hierarchical clustering of the expression profiles of 86 LcERFs in various tissues. The FPKM value was normalized by the Z-score algorithm. (**B**) Relative expression of five potential genes in various tissues. The round dot is the original value, and the error bar shows Std. deviation. For statistical analyses, ANOVA and Duncan tests were used (*p* < 0.05)

We investigated the expression profiles of LcERF genes in various tissues by Illumina RNA-Seq data [31] and constructed a heatmap, revealing that 86 LcERF genes were detected in the various tissues, including 34 genes in the DREB subfamily, 35 genes in the ERF subfamily, 12 genes in the AP2 subfamily, 4 genes in the RAV subfamily, and one Soloist gene. To explore the differential expression of these genes in different tissues, the FPKM values were standardized by row with TBtools software. Then, the standardized results were clustered by row and column (Fig. 4A). The results showed that several genes were expressed in all tissues and clustered in a large group. In addition, the column cluster divided the other genes based on their different expression patterns, including pistil-specific, stamen-specific, leaf-specific, shoot-specific, and other patterns.

### Expression patterns of LcERF genes and discovery of shoot-specific genes

**Fig. 5:**
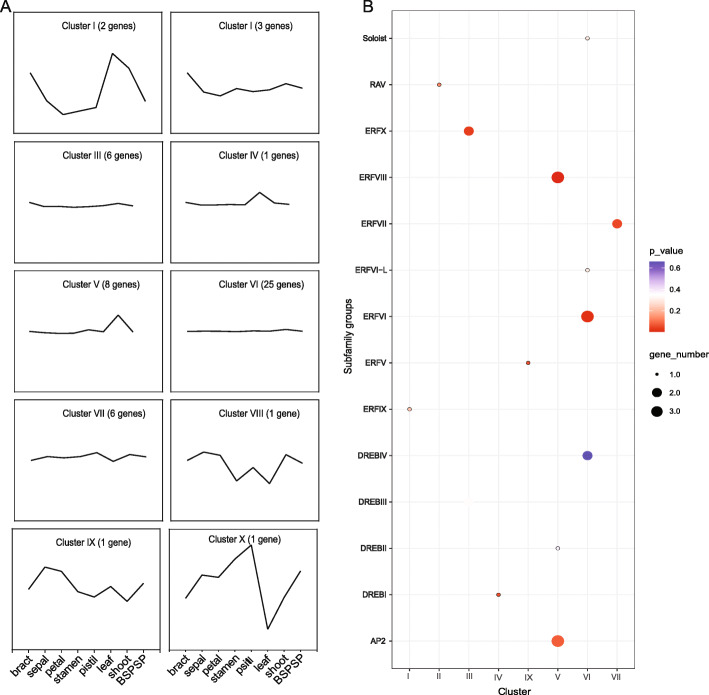
Series test of cluster analysis of *LcERF* genes. (**A**) Ten clusters of all 86 LcERFs using the K-means method. The black line represents the trend of gene expression in different tissues. (**B**) Cluster detection of different subfamilies of LcERFs. The size of the circle represents the number of genes, and the color represents the *p*-value

To reveal genes involved in shoot and leaf development, we intentionally focused on genes that were expressed specifically in the shoot tissue. All the *LcERF* family genes were divided into ten clusters in accordance with the K-means method in STEM software. Accordingly, cluster IV and cluster V showed tissue-specific expression in leaves and shoots, respectively. Cluster V contained eight genes, while cluster IV contained only one (Fig. 5A). We further tested the results of STC analysis of all the LcAP2/ERF groups with an adjusted *p*-value (*p* ≤ 0.05), and only six of eight genes were significantly clustered in the V cluster, while the single member of cluster IV failed to pass the significance test (Fig. 5B). Among these six genes, three are ERF VIII members (*LcERF57*, *LcERF58,* and *LcERF63*), and another three belong to the AP2 subfamily (*LcERF94*, *LcERF96,* and *LcERF98*).

The expression of the six candidate genes showing shoot-specific patterns was further verified using RT-qPCR. We determined the expression of these six genes in seven tissues: leaf, shoot, sepal, petal, stamen, pistil, and stem. Consistent with the STC results, the *LcERF94*, *LcERF96,* and *LcERF98* genes were primarily expressed in shoots. While the expression of *LcERF63* in different tissues was not specific, it was detected in both shoots and flowers, and *LcERF58* was detected in all tissues (Fig. 4B). Unexpectedly, we failed to detect the *LcERF57* genes even with repetitive optimization of the primer design and amplification conditions; hence, we did not perform the following assay of this gene. Considering the potential functions annotated in the NCBI GenBank and Gene Ontology (GO) databases, dual roles may exist for these three genes in Group VIII, as we inferred. Moreover, this conjecture has been clarified in previous studies, which have confirmed that ERF VIII subgroup genes play an important role in vitro shoot regeneration and development [32].

We then annotated the functions of these six genes by submitting sequences to TAIR (The Arabidopsis Information Resource). Through alignment and annotation, all three genes of the AP2 subfamily were annotated as the *AINTEGUMENTA* (*ANT*) or *AINTEGUMENTA-*like (*AIL*) gene, which is also considered to be involved in maintenance of the shoot apical meristem (GO:0010492), the auxin-mediated signaling pathway involved in phyllotactic patterning (GO:0060774), plant organ morphogenesis (GO:1905392), cell division (GO:0051301), and cell growth (GO:0016049). However, *LcERF57*, *LcERF58,* and *LcERF63* from the VIII group were annotated with different functions, such as negative regulation of the ethylene-activated signaling pathway (GO:0010105, GO:0009873) and glucosinolate metabolic process (GO:0019760).

### Potential roles of shoot-specific candidates involved in shoot and leaf development

**Fig. 6:**
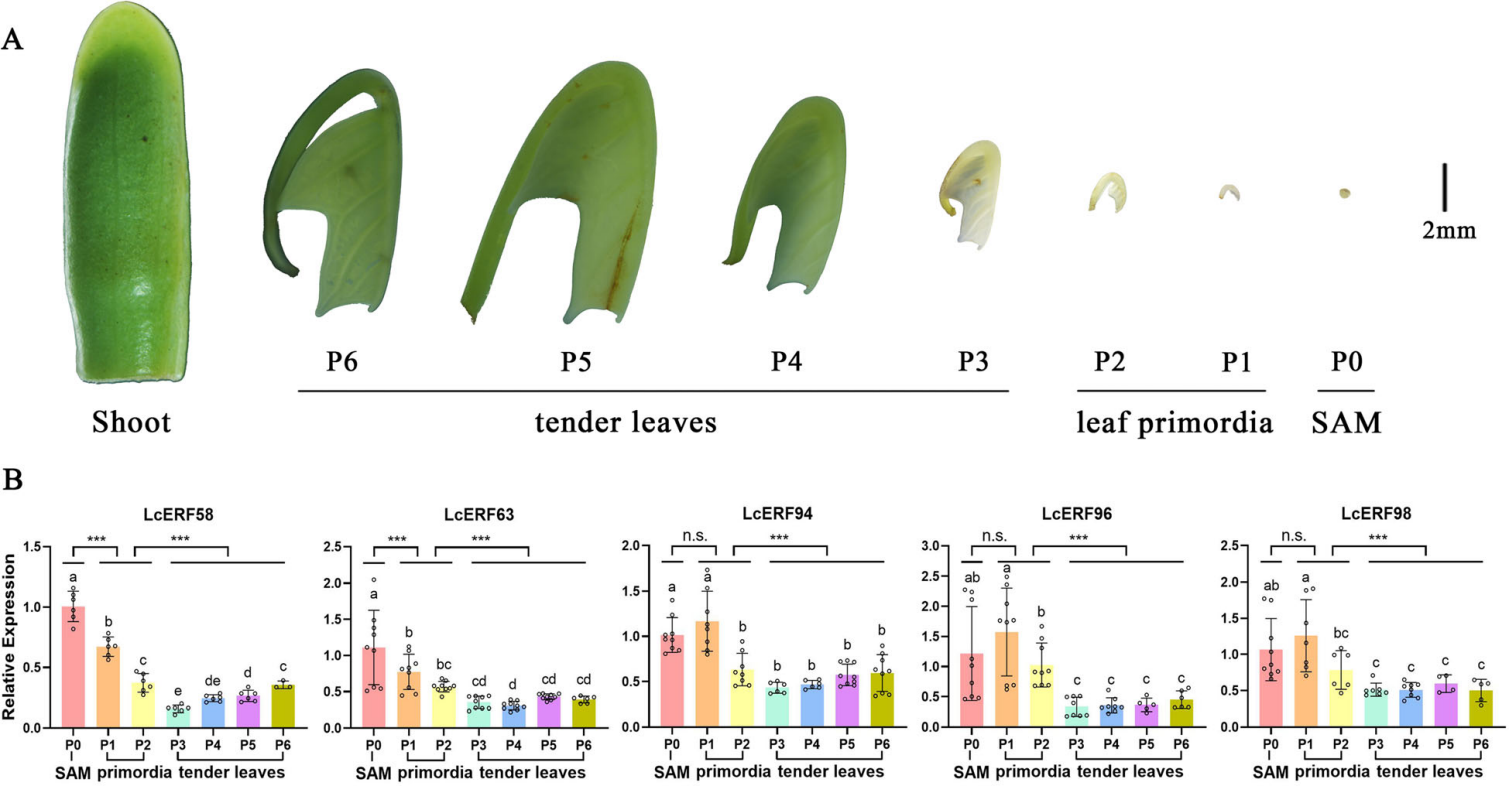
Dynamic changes in *L. chinense* leaves at different developmental stages. (**A**) Morphological anatomy of the shoots under the stereomicroscope. According to the anatomic structure, the developmental stages of leaf were defined as P0 (SAM), P1, P2, P3, P4, P5, and P6 (P1 ~ P2 were the leaf primordia, while P3 ~ P6 were identified as tender leaves). (**B**) Relative expression of five candidates in different developmental stages. The round dot is the original value, and the error bar shows Std. deviation. For statistical analyses, ANOVA and Duncan tests were used (*p* < 0.05). (*** indicates *P*-value < 0.001; n.s. indicates no significant)

It is generally known that young leaves rise from shoots, and we further dissected the anatomical structure of the shoot layer by layer. In vitro shoots were divided into multiple layers of tender leaves, and discontinuous developmental stages of leaves, i.e.*,* P0 (SAM), P_1_, P_2_, P_3_, P_4_, P_5_, and P_6_, are displayed in Fig. 6A. Referring to the nomenclature, P1 and P2 periods were identified as leaf primordia, while P3 ~ P6 were tender leaves when checking the anatomical structure. Leaf morphology and leaf lobes formed during the P1 and P2 stages, and the leaf size gradually increased from P3 to P6 (tender leaves), while SAM was the primary contributor to primordium differentiation in *L. chinense* leaves.

Attracted by the shoot-specific patterns of the candidate genes, we further explored the potential functions of the candidates by detecting the expression from P0 to P6. RT-qPCR was performed to detect the expression of target genes from P1 to P6 as well as SAM (Fig. 6B). The results revealed that the expression of these genes decreased gradually from P0 to P6. Specifically, a significant difference was detected in the expression of the leaf primordia (P1-P2) and tender leaves (P3-P6) when examining the *LcERF94*, *LcERF96*, and *LcERF98* genes; however, adverse consequences were found in comparisons of the SAM (P0) and leaf primordia (P1-P2). Taken together, this evidence supports *LcERF94/96/98* genes as potential candidates involved in the early-stage development of leaf morphology in *L. chinense*.

### Subcellular localization of LcERF genes

**Fig. 7:**
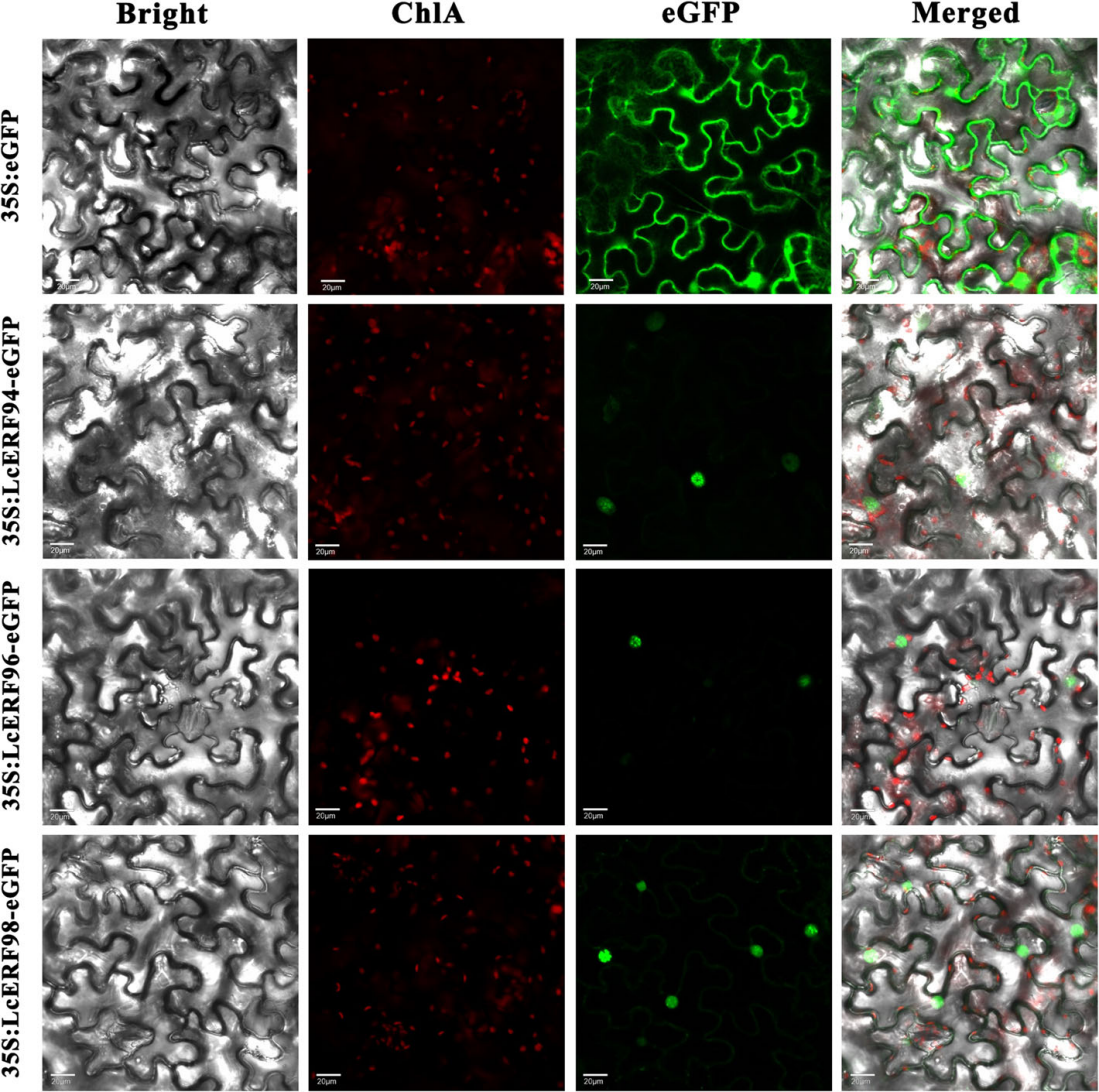
Subcellular localization of *LcERF94/96/98* proteins. The candidates were driven by the 35S promoter. The excitation maximum of GFP was 488 nm, and the excitation maximum of GFP was 509 nm

To investigate the potential function of these AP2 genes in transcriptional regulation, we detected the subcellular localization of *LcERF94/96/98* using young tobacco leaves. Confocal microscopy was used to observe and photograph the transient transformed lower epidermal cells of tobacco leaves, and visible, GFP fluorescence, chlorophyll fluorescence, and merged field images were obtained (Fig. 7). The *35S::GFP*, as a control sample, showed GFP fluorescence in the whole cell. The GFP fluorescence of pBI121-*35S::GFP -LcERF94/96/98* was observed only in the nucleus, which is consistent with the characteristics of TFs, and these histological observations indicated that *LcERF94/96/98* might function as TFs in the nucleus.

## Discussion

The AP2/ERF transcription factors are ubiquitous in plant species and act as vital regulators governing various biological processes. They have been studied in many species, and the total number of AP2/ERF family genes varies among different plants, for example, 147 in *A. thaliana* [18, 19], 163 in rice [19, 21], 132 in grape [33], and 200 in *Populus trichocarpa* [34] et al. [14, 35–38]. Based on the features of the conserved domain, the AP2/ERF TFs are classified into four groups as ERF, AP2, RAV and soloist. Global survey of the ERF subfamily was first reported by Sakuma, in which all AtERFs were divided into DREB (group A) and ERF (group B) based on the sequence identities of the DNA-binding domains [18]. Furthermore, Nakano developed Sakuma’s classification and subdivided ERF genes into 12 groups according to the genome annotation and phylogenetic analysis of *A. thaliana* and rice [19]. Similarly, in this study, all 104 LcAP2/ERFs were wholly sorted into four subfamilies: ERF, AP2, RAV and soloist, which is consistent with former classifications. Meanwhile, each subfamily of AP2/ERF was provided with an unequal number of proteins in distinct species, while in any case, the ERF subfamily was the largest one, and both the DREB group and the ERF group are of particular interest owing to their involvement in plant responses to stresses [39].

Gene function is mainly dependent on unique features [40]. The “WLG” (Trp-28, Leu-29, and Gly-30) of the AP2/ERF domain is completely conserved in the DREB and ERF subfamilies, and it has also been proven in many other plants, such as Arabidopsis [19], rice [21]^,^ and sorghum [41]. However, the major differentiators of ERF and DREB subfamilies are nonsynonymous substitutions at some specific motifs, i.e., V14 (valine) and E19 (glutamic) are conserved in DREB, while A14 (alanine) and D19 (aspartic) exist in ERF [18]. In many cases, ERF and DREB genes play a role in response to stresses such as drought, salt and low temperature through ABA-independent signaling pathways. DREB proteins, such as *CBF1*, *CBF2*, *CBF3* [42–44] and DREB1B [45], contain the DNA binding domain that binds to the *cis*-acting dehydration-responsive element DRE/CRT in the promoters and activates its expression to enhance the abiotic resistance of transgenic plants [46, 47]. While the ERF subfamily genes mainly work to mediate pathogen- and disease-related stimuli by integrating multiple signaling pathways, such as the jasmonic acid (JA), ethylene (ET) and salicylic acid (SA) pathways [39]. Evidence shows that ERF proteins bind to the cis-element GCC box (GCCGCC) and directly regulate the expression of pathogenesis-related (PR) genes [48]. However, exceptions also exist that the ERF VII, VIII, and IX groups partially respond to ethylene signal to participate in plant growth and development. For instance, *MaERFs* are involved in regulating ethylene-mediated fruit ripening of banana [49]. Recent research on pineapple also showed that *AcERFs* might be positive and key regulators in response to ethylene and induce flowering in pineapple [50], which provided evidence that some *ERF* genes might be involved in the development of floral organs.

In contrast to ERFs, AP2 subfamily members are considered regulators that help to maintain meristems and regulate organ initiation and growth [51]. Although containing the AP2 domain as well, the AP2 proteins are quite different from the ERFs in terms of conserved domains and gene structure. Conserved motif analysis presented that the amino acid structure of the AP2 domain displayed a low similarity between the AP2 and ERF subfamilies, and “WLG” was converted into “YLG” elements in all AP2 subfamily members [14]. Furthermore, the AP2 genes were interrupted by 6 ~ 11 introns, which also gives them a variety of functions in the plant development process [16]. In most cases, the AP2 genes respond to plant development and morphogenesis, especially in hormone-mediated morphological development of leaf and flower organs [52–54]. *AINTEGUMENTA* (*ANT*) and *AINTEGUMENTA-LIKE* (*AIL*) TFs of the AP2 subfamily are expressed in all dividing tissues and have central roles in developmental processes such as embryogenesis [55], root development [56], organ initiation, and growth [57].

Plant leaves are initiated from the shoot apical meristem (SAM), and later grow toward three axes, including the adaxial-abaxial (top-bottom), proximal-distal (base-to-tip) and medio-lateral (central-to-edge) axes [58]. Once the polarity is established, the leaf primordium starts to extend to all sides and finally determines leaf size and shape [59]. In this period, multiple TFs were up-regulated and involved in morphogenesis, such as MYB-domain proteins, *KNOTTED1*-like homeobox proteins, *AUXIN RESPONSE FACTORS*, *HD-ZIP III* genes, basic helix-loop-helix (bHLH) transcription factors and *AP2/ERF*, etc. Overexpressed *Larix AP2L1* in *Arabidopsis* leads to enlarged rosette leaves by affecting cell size as well as cell proliferation [60]. The AP2 genes were also involved in the arrangement of phyllotaxy. The *PLT3*, *PLT5* and *PLT7* genes acted as key regulators that reshaped the phyllotaxis pattern of *A. thaliana* by regulating *AtPIN1* activity [61]. Subsequent research further showed that *PLT5* and *PLT7* were differentially expressed between *C. hirsuta* and *A. thaliana* during early leaf development, and expressed *ChPLT7* in the simple leaf margin of *A. thaliana* under the *CUC2* promoter to cause ectopic leaflet formation [62].

In this study, *LcERF94*, *LcERF96*, and *LcERF98* showed high similarity with *ANT* or *AIL* in their conserved domain and gene structure. These three candidates presented high expression levels in early leaf development, and these patterns were consistent with the patterns of *ANT* and *AIL* genes from *Arabidopsis* and *Cucurbita moschata* [57, 63, 64]. Based on these results, we conjecture that three candidates (*LcERF94*, *LcERF96*, and *LcERF98*) might regulate early leaf development in the same way as the *ANT* and *AIL* genes. Among them, *ANT* genes exhibit the highest expression at the top of shoots, and participate in the initiation, growth and organ size development of higher plant organs by regulating cell proliferation and division [57]. Additionally, *AIL* genes were detected with high levels in young and actively dividing tissues in *Arabidopsis* by detecting the relative expression of various tissues [63]. More importantly, after receiving stimulation from growth regulators, the *ANTs* are regulated positively to maintain cell meristematic competence, thereby modulating the expression of cell growth regulators and promoting organ growth [57].

## Conclusions

In summary, we identified 104 putative AP2/ERF genes in *Liriodendron,* which were entirely grouped into four major subfamilies. The classification and expression profiling of the AP2/ERF genes provide information for the identification of potential target genes, allowing conjecture regarding the roles of these genes. Furthermore, the STC analysis and dynamic patterns of *LcERF94/96/98* in leaf development period indicated that these genes might affect shoot and leaf development. Regardless, this study provides a new perspective for exploring the function of LcERFs in regulating plant growth and development, and three genes identified in this study are good candidates for subsequent functional investigations.

## Methods

### Plant materials

Plant materials were collected from the forest farm attached to Nanjing Forestry University, Jiangsu Province, China (119°13′20″E, 32°7′8″N). Sample trees were originally from Lushan, Jiangxi Province (116°0′E, 29°32′N) (Specimen No. 20010020016, deposited in the specimen room of Nanjing Forestry University) and have been planted in forest farm since 1993. In the middle of spring 2018, various tissues, including shoots, leaf sepal, petal, stamen, pistil, and stem tissues, were removed from mature *L. chinense* trees. In addition, young leaves in distinct developmental periods were also collected from the same trees in the summer of 2019. For each of the samples, no less than three biological replicates were collected, all of which were removed, immediately frozen in liquid nitrogen, and stored at − 80 °C.

### Identification and characteristics of the AP2/ERF genes of *L. chinense*

The *L. chinense* genome, which was published in 2019, was downloaded from the NCBI genome resource database [29]. Arabidopsis AP2/ERF proteins were obtained from the Plant Transcription Factor database (http://plntfdb.bio.uni-potsdam.de/v3.0/) and utilized for BLASTP searches against genome sequences of *L. chinense* with expected values less than 10^− 3^. Moreover, the AP2/ERF domain (Pfam accession is PF00847) was downloaded from the Pfam entrance database (http://pfam.xfam.org/) and then used to retrieve AP2/ERF-domain amino acid sequences from all annotated genes of the *L. chinense* genome using the HMMER program (v3.0). In addition to the genome-wide survey, we also used the published transcriptome data (No. SRR8101043, SRR8101042, SRR8101041 and SRR8101040) to determine the integrity and reliability of all AP2/ERF information as much as possible [30].

All candidate AP2/ERF-domain amino acid sequences were assessed based on the presence of the conserved domain using Pfam search (http://pfam.xfam.org/search#tabview=tab1) and CDD search (https://www.ncbi.nlm.nih.gov/Structure/bwrpsb/bwrpsb.cgi) procedures. In addition, sequences that incorrectly occupied or even did not carry a complete domain were removed from the list of putative genes. Conserved motifs are essential characteristics of a gene family that perform various functions. Thus, all putative AP2/ERF-domain amino acid sequences were divided into distinct subfamilies according to their motif characteristics.

### Phylogenetic analysis

To determine the evolutionary relationship of all AP2/ERF sequences, subfamily classification of all AP2/ERF sequences was further confirmed by constructing an unrooted phylogenetic tree with the neighbor-joining (NJ) method. The conserved domain extracted from the whole length of the AP2/ERF sequences was used for multiple sequence alignment using ClustalW with default parameters in the MEGA X software package [65]. The phylogenetic tree was then constructed using the NJ method with 1000 bootstrap replicates in MEGA X. Finally, the network profile of the phylogenetic tree was visualized using Evoview online software (https://www.evolgenius.info/evolview/#login).

### Conserved motif and gene structure analyses

To investigate the gene structure of the AP2/ERF family, the annotation profile of the *L. chinense* genome was retrieved from the China National GeneBank (CNGB). Information on all exon and intron loci was extracted and later visualized with GSDS (v2.0) (http://gsds.cbi.pku.edu.cn/). In parallel with the gene structure surveys, the conserved motifs of the *L. chinense* AP2/ERF family were predicted utilizing MEME (v5.1.1) (http://meme-suite.org/tools/meme) based on the full-length protein sequences with the following parameters: maximum number of motifs of 15; motif sites distributed among sequences with zero or one per sequence model. The results of the motif analysis were then visualized with TBtools software [66].

### Series test of cluster (STC) analysis of LcAP2/ERF expression in various tissues

The expression profiles of various tissues (including bract, sepal, petal, stamen, pistil leaf, shoot, and a mixture of all the floral organs named BSPSP) were obtained from the NCBI database (No. PRJNA559687) and used to demonstrate the expression profiles of all LcAP2/ERF genes [31]. The data preprocessing of sequences was performed mainly in three steps: 3′-trimming, filtering by Phred score and removing low complexity sequences (less than Q20) using the FastQC toolkit. All the filtered clean reads were assigned to the reference genome of *Liriodendron chinense* using TopHat2 [67]. Programs were run with defaults options except for a maximum size of intron of 5000 base pair. Expression of each gene was further calculated using the fragments per kilobase of exon model per million mapped fragments (FPKM) measurements by Cufflinks [68]. The arithmetic used the default parameters (cufflinks SRR9945430.bam -G genes.gtf -o FPKM.result) [69]. The FPKM values were standardized by Z-score normalization to reflect the expression pattern of all annotated LcAP2/ERF genes. Furthermore, we generated heat maps of all genes using TBtools according to the instructions (parameters: Log Scale, Base 2.0, LogWith 1.0; cluster by row and column) [66].

To discover the genes involved in shoot development, we used STC analysis (STC, series test of cluster) to derive the candidates that were markedly enriched in the shoot-specific cluster. STC analysis was performed using K-means clustering method by STEM program, the number of clusters (K) was 10 and number of random starts was 20. We filter expression values with default parameters, and the correction method was Bonferroni’s correction with the significant level was 0.05 [70]. Then cluster analysis and functional annotation of these candidates were performed in GenBank and the TAIR database.

### Histological anatomy and morphological observation

We collected and dissected the shoots in layers to obtain tender leaves at different stages of development and observed them under an OLYMPUS SZX16 stereomicroscope. The developmental stages of leaf were defined as P0 (SAM), P1, P2, P3, P4, P5, and P6 from the innermost to the outermost layer of the buds based on their developmental morphology (P1 ~ P2 were the leaf primordia, while P3 ~ P6 were identified as tender leaves). Moreover, materials from different stages were collected and immediately frozen in liquid nitrogen and stored at − 80 °C for RNA extraction.

### RNA extraction and RT-qPCR analysis of AP2/ERF genes

Total RNA was extracted from samples with the RNAprep pure kit (Tiangen, Beijing, China) according to the manufacturer’s instructions. Then, cDNA was synthesized from 500 ng of total RNA using PrimeScirptTM RT Master Mix (TaKaRa, Dalian, China) in a 10-μL reaction volume according to the instructions. Before polymerase chain reaction, the cDNA was diluted 1/10 with mother liquor made with deionized water to reduce systematic error. Then, specific primers for the *LcERF* genes were designed with Oligo 7.0 software adhering to the instructions (see Additional file 2: Table S2). RT-qPCR was performed on a StepOnePlus™ System (Applied Biosystems) with 10-μL reaction mixtures containing 5 μL of 2× SYBR Premix Ex Taq, 0.2 μL of 50× ROX Reference Dye (TaKaRa, Dalian, China), primers, and cDNA. The relative expression of the *LcERF* genes was calculated using the ΔΔC_T_ method. Three biological replicates were set per sample, and each sample had three technical replicates. In addition, the *LcActin97* gene was used as a reference gene in this process, which has been shown to be reliable in *L. chinense* [71]. In addition, all the RT-qPCR relative expression data were further analyzed using SPSS 11.5, and ANOVA and Duncan’s test were also performed to test the significant differences among various samples.

### Subcellular localization assay

We further constructed recombinant proteins that fused the eGFP marker with the LcERF proteins in the C-terminus. The pBI121-eGFP vector (GUS was replaced with GFP in the original PBI121 vector) was restricted by the XbarI and BamHI enzymes along with insertion of the recombinant proteins into the digested vector. Subsequently, the recombinant plasmid sequences were verified by the Sanger sequencing platform and then transferred into *Agrobacterium tumefaciens* (GV3101). After overnight incubation at 28 °C, the OD600 value of the bacterial solution reached 0.6 ~ 0.8. We collected recombinant bacteria by centrifugation at 4000 rpm and resuspended the bacteria in infection buffer (10 mM MgCl_2_, 10 mM MES, 150 μM HO-AS, with a final pH = 5.6). Moreover, the helper vector P19 was subjected to the same treatment and mixed equally with ERF. The resuspended mixtures were injected into tender tobacco leaves. After 1 ~ 2 days of coculture, we observed and recorded the GFP fluorescence signal under a laser confocal microscope.

## Supplementary Information

**Additional file 1: Table S1.** List of the 104 AP2/ERF genes identified in *L. chinense.*

**Additional file 2: Table S2.** List of primer sequences in this study.

## Data Availability

All data presented in this study are provided either in the manuscript or additional files. The datasets generated and analysed during the current study are available in the NCBI with the accession numbers SRR8101043, SRR8101042, SRR8101041, SRR8101040 and PRJNA559687.

## Abbreviations

TFs: Transcription factors
AP2/EREBPs: APETALA2/ethylene responsive element-binding proteins
ERF: Ethylene responsive element binding factor
DREB: Dehydration responsive element binding factor
RAV: Related to ABI3/VP
ANT: AINTEGUMENTA
AIL: AINTEGUMENTA-Like
SAM: Shoot Apical Meristem
DRN: DORNROESCHEN
DRNL: DRN-LIKE
FPKM: Fragments Per Kilobase Million
GFP: Green fluorescent protein
